# On-treatment lung immune prognostic index is predictive for first-line PD-1 inhibitor combined with chemotherapy in patients with non-small cell lung cancer

**DOI:** 10.3389/fimmu.2023.1173025

**Published:** 2023-05-25

**Authors:** Anning Xiong, Jianlin Xu, Shuyuan Wang, Runbo Zhong, Jun Lu, Tianqing Chu, Wei Zhang, Ying Li, Xiaoxuan Zheng, Baohui Han, Wei Nie, Hua Zhong, Xueyan Zhang

**Affiliations:** Department of Respiratory and Critical Care Medicine, Shanghai Chest Hospital, Shanghai Jiao Tong University School of Medicine, Shanghai, China

**Keywords:** lung immune prognostic index, peripheral blood cells, immune-checkpoint inhibitors, combination chemotherapy, non-small cell lung cancer

## Abstract

**Background:**

Inflammation is a factor that promotes tumor progression and immunosuppression. Lung immune prognostic index (LIPI) is a non-invasive and easily calculated indicator of inflammation. This study aimed to investigate whether continuous assessment of LIPI has predictive value for chemoimmunotherapy in non-small cell lung cancer (NSCLC) patients receiving first-line programmed cell death 1 (PD-1) inhibitor plus chemotherapy. In addition, the predictive value of LIPI in patients with the negative or low programmed death-ligand (PD-L1) expression level was also explored.

**Methods:**

Totally, 146 stage IIIB to IV or recurrent NSCLC patients who received first-line PD-1 inhibitor combined with chemotherapy were enrolled in this study. The LIPI scores were calculated at baseline (PRE-LIPI) and after two cycles of the combined administration (POST-LIPI). This study analyzed the relationship between good/intermediate/poor PRE (POST)-LIPI and objective response rate (ORR), as well as progression-free survival (PFS) using logistic and Cox regression models. In addition, the predictive value of LIPI in patients with the negative or low PD-L1 expression level was explored. To further assess the potential predictive value of continuous assessment of LIPI, the association of sum (LIPI) [sum(LIPI) = PRE-LIPI + POST-LIPI] and PFS was analyzed in the 146 patients.

**Results:**

Compared with good POST-LIPI group, significantly lower ORRs were found in intermediate POST-LIPI (P = 0.005) and poor POST-LIPI (P = 0.018) groups. Moreover, intermediate POST-LIPI (P =0.003) and poor POST-LIPI (P < 0.001) were significantly associated with a shorter PFS than good POST-LIPI. Additionally, a higher POST-LIPI score was still significantly associated with poorer treatment efficacy in patients with the negative or low PD-L1 expression level. Moreover, a higher sum (LIPI) score was significantly correlated with a shorter PFS (P = 0.001).

**Conclusion:**

Continuous assessment of LIPI might be an effective method for predicting the efficacy of PD-1 inhibitor plus chemotherapy in NSCLC patients. In addition, in patients with the negative or low PD-L1 expression level, it might also have a potential predictive value for therapeutic efficacy to continuously assess LIPI during the treatment.

## 1 Introduction

The immune checkpoint inhibitors (ICIs) blocking programmed cell death 1 (PD-1) represent an unprecedented breakthrough in the treatment of advanced non-small cell lung cancer (NSCLC) (1). However, the clinical benefits of PD-1 inhibitors are more remarkable in patients with the high programmed death-ligand 1 (PD-L1) expression level [tumor proportion score (TPS) ≥ 50%]. To increase the proportion of responders, the combination of ICIs and chemotherapy has been suggested and become a standard therapy for NSCLC (2, 3) patients without sensitive driver mutations. Clinical outcomes have been improved after the combination of immunotherapy and chemotherapy in those NSCLC patients (4–7). The combination therapy is more frequently used than immune monotherapy in the real world.

To find patients who are most likely to benefit from immunotherapy, several biomarkers have been explored (8–13). The predictive value of PD-L1 expression level has been validated in immunotherapy (14). Nevertheless, as there is a heterogeneity in PD-L1 expression level (15, 16), the time and the site of sampling may affect the results. Blood tumor mutational burden (bTMB) can be detected by blood and this index has a certain predictive effect on the efficacy of immunotherapy (17). However, the relationship between bTMB and the prognosis of patients receiving immunotherapy has been reported as nonlinear (18), and this might indicate why it is difficult to determine the cutoff value of bTMB. Furthermore, chemoimmunotherapy is frequently used in patients with the negative or low PD-L1 expression level and without driver mutations in the real world, which is not the population with the most remarkable benefit among patients receiving ICI plus chemotherapy (6, 19). Therefore, there is an urgent need to explore valid and easily accessible predictors for the combination therapy.

Inflammation has been recognized as a contributor to cancer progression and one of the causes of immunoresistance in patients with cancer (20, 21). Neutrophils participate in systemic inflammatory response and cancer progression (22). Lactate dehydrogenase (LDH), another indicator of inflammation, plays a role in reflecting the patient’s tumor load (23). To establish a biomarker with a great predictive value, Mezquita et al. developed a lung immune prognostic index (LIPI) according to derived neutrophil to lymphocyte ratio [dNLR; neutrophils/(leukocytes – neutrophils)] higher than 3 and LDH level greater than the upper limit of normal (ULN) (24). LIPI is a blood-based index that possesses the advantages of being convenient and easy to measure. Therefore, it has the feasibility of dynamic monitoring during the treatment. However, previous studies have mainly explored the predictive value of LIPI at baseline (25, 26), and the relationship between LIPI in the early-stage treatment and patient prognosis has rarely been analyzed.

Thus, the present study aimed to evaluate the predictive value of continuous assessment of LIPI in the early-stage of treatment of NSCLC patients treated with a first-line PD-1 inhibitor plus chemotherapy. Furthermore, it was attempted to explore whether LIPI had predictive value in patients with the negative or low PD-L1 expression level.

## 2 Methods

### 2.1 Patients

We conducted a retrospective study of stage IIIB to IV or recurrent NSCLC patients who received a first-line PD-1 inhibitor plus cytotoxic chemotherapy at Shanghai Chest Hospital from January 2019 to June 2021. The inclusion criteria were as follows: (1) patients who were diagnosed with NSCLC pathologically; (2) patients with stage IIIB to IV or recurrent NSCLC according to the eighth edition of the tumor, node, metastasis (TNM) classification for lung cancer; (3) patients who received a first-line PD-1 inhibitor combined with chemotherapy.

The exclusion criteria were as follows: (1) patients who had sensitizing epidermal growth factor receptor (EGFR)/anaplastic lymphoma kinase (ALK)/c-ros oncogene 1 receptor tyrosine kinase (ROS1) mutations; (2) patients who received ICI-based neoadjuvant therapy; (3) exposure to infection or antibiotics within 7 days before the blood test using for calculating LIPI score; (4) unavailability of data related to the LIPI score and other parameters with potential influence on the efficacy of the combination therapy.

The end of the follow-up period was August 31st, 2022. This study was approved by the ethics committee and institutional review board of Shanghai Chest Hospital (Shanghai, China).

### 2.2 Study design

The clinical characteristics, including age, gender, smoking history, Eastern Cooperative Oncology Group performance status (ECOG PS), histology, TNM stage, PD-L1 expression level, radiotherapy, immune-related adverse events (irAEs), albumin (ALB) level, leukocyte count, neutrophil count, and LDH level were extracted from the patients’ medical records.

Complete blood cell count, and LDH and ALB levels were extracted at baseline (within 10 days before the combination treatment) and before the third administration cycle (within 1 day before the third injection). The value of dNLR was calculated as follows: neutrophil count/(leukocyte count – neutrophil count). PRE-LIPI and POST-LIPI were defined according to dNLR larger than 3 and LDH level higher than ULN at baseline and after two cycles of the treatment, respectively. If patients had disease progression before the second administration, POST-LIPI was calculated according to the blood test result, which was the closest to the first radiological assessment. Patients were classified into three groups: good PRE (POST)-LIPI group (0 factor), intermediate PRE (POST)-LIPI group (1 factor), and poor PRE (POST)-LIPI group (2 factors) (24). ALB level was dichotomized at the upper limit of hematological test based on clinical significance. Sum (LIPI) was calculated as follows: sum (LIPI) = PRE-LIPI + POST-LIPI.

PD-L1 expression level was assessed by immunohistochemistry using tumor tissue or biopsy specimen. The PD-L1 expression level was measured using TPS, which is the percentage of viable tumor cells revealing partial or complete membrane staining at any intensity. A PD-L1 TPS of 1% or higher was considered positive.

### 2.3 Treatment and evaluation criteria

All patients underwent either chest computed tomography (CT), bone scan and abdominal ultrasound examinations or positron emission tomography (PET)-CT in lieu of the above examinations prior to the initiation of combination treatment. All patients underwent brain magnetic resonance imaging (MRI). Patients were regularly followed up. Chest CT, abdominal ultrasound, and brain MRI were performed every 2 months during the treatment. Bone scan was carried out once a year. Furthermore, the examinations were conducted if patients developed corresponding symptoms.

Pembrolizumab (200 mg every three weeks), sintilimab (200 mg every three weeks) or tislelizumab (200 mg every three weeks) were intravenously administrated. The choice of combination treatment was determined by physicians, while other drugs included pemetrexed, docetaxel, gemcitabine, paclitaxel/nab-paclitaxel/paclitaxel liposome, vinorelbine, carboplatin, and cisplatin.

The primary endpoint was progression-free survival (PFS), which was defined as the interval from the first administration cycle of treatment to disease progression. The secondary endpoint was objective response rate (ORR), which was defined as complete response plus partial response. Tumor response was determined by a radiologist and a clinician independently based on the Response Evaluation Criteria in Solid Tumors (ver. 1.1).

### 2.4 Statistical analysis

The correlations of LIPI and the patient’s characteristics as well as ORR were analyzed using χ2 test or Fisher’s exact test. Median PFS was derived using the Kaplan-Meier method. Cox regression analysis was performed to analyze hazard ratio (HR) of various factors for PFS. Logistic regression analysis was carried out to evaluate influences of the factors on ORR. The statistical significance was defined as *P* < 0.05 (two-sided). SPSS 24.0 (IBM, Armonk, NY, USA) and GraphPad Prism 8.0 (GraphPad Software Inc., San Diego, CA, USA) software were used for the statistical analysis. Receiver operating characteristic (ROC) curves and related analyses were finished by using R version 4.2.3.

## 3 Results

### 3.1 Patients’ characteristics

In total, 146 eligible NSCLC patients who received first-line PD-1 inhibitor-based combination therapy were included and further analyzed in this study (**Supplementary Figure 1**).

In this study, median follow-up time was 21.0 months [95% confidence interval (CI), 19.4-22.6] and median PFS time was 9.0 (95% CI, 6.9-11.1). Patients’ baseline characteristics are listed in **Table 1**.

**Table 1:** Baseline characteristics.

Characteristic	Total (n = 146)
Age, n (%)	
<65	72 (49.3)
≥65	74 (50.7)
Sex, n (%)	
Male	118 (80.8)
Female	28 (19.2)
Smoking history, n (%)	
Never	36 (24.7)
Current/former	110 (75.3)
ECOG PS	
0-1	142 (97.3)
≥ 2	4 (2.7)
Histology, n (%)	
Squamous	49 (33.6)
Non-squamous*	78 (53.4)
NOS	19 (13.0)
T stage, n (%)	
0-2	95 (65.1)
3-4	51 (34.9)
N stage, n (%)	
0-2	56 (38.4)
3	90 (61.6)
TNM stage, n (%)	
III B/III C	34 (23.3)
IV/Recurrent	112 (76.7)
PD-L1 TPS, n (%)	
TPS < 1%	46 (31.5)
1% ≤ TPS ≤ 49%	45 (30.8)
TPS ≥ 50%	41 (28.1)
Not evaluable	14 (9.6)

*Non-squamous tumor included adenocarcinoma and lymphoepithelioma-like carcinoma.

ECOG PS, Eastern Cooperative Oncology Group performance status; NOS, not otherwise specified; PD-L1, programmed cell death-protein 1; TPS, tumor proportion score.

Among them, 66 (45.2%) patients were classified into good PRE-LIPI group, 60 (41.1%) patients were in intermediate PRE-LIPI group, and the remaining 20 (13.7%) patients were in poor PRE-LIPI group. In addition, when patients were classified according to their POST-LIPI scores, 83 (56.8%) patients were in good POST-LIPI group, 54 (37.0%) patients were in intermediate POST-LIPI group, and the remaining 9 (6.2%) patients were in poor POST-LIPI group. Patients’ characteristics were balanced among the three PRE(POST)-LIPI groups except for ALB level. A higher LIPI score was significantly correlated with a low ALB level both at baseline (*P* = 0.042) and in the early-stage (*P* = 0.042) of treatment (**Supplementary Table 1** and **Table 2**).

**Table 2:** The correlation of POST-LIPI with other patients’ characteristics.

Characteristic	Good POST-LIPI (n = 83)	Intermediate POST-LIPI (n = 54)	Poor POST-LIPI (n = 9)	*P*
Age, n (%)				
<65	40 (48.2)	30 (55.6)	2 (22.2)	0.177
≥65	43 (51.8)	24 (44.4)	7 (77.8)	
Sex, n (%)				
Male	63 (75.9)	46 (85.2)	9 (100)	0.163
Female	20 (24.1)	8 (14.8)	0 (0)	
Smoking history, n (%)				
Never	24 (28.9)	11 (20.4)	1 (11.1)	0.440
Former/Current	59 (71.1)	43 (79.6)	8 (88.9)	
ECOG PS				
0-1	82 (98.8)	52 (96.3)	8 (88.9)	0.153
≥ 2	1 (1.2)	2 (3.7)	1 (11.1)	
Histology, n (%)				
Squamous	27 (32.5)	19 (35.2)	3 (33.3)	0.761
Non-squamous*	47 (56.6)	27 (50.0)	4 (44.4)	
NOS	9 (10.8)	8 (14.8)	2 (22.2)	
T stage, n (%)				
0-2	52 (62.7)	38 (70.4)	5 (55.6)	0.495
3-4	31 (37.3)	16 (29.6)	4 (44.4)	
N stage, n (%)				
0-2	30 (36.1)	23 (42.6)	3 (33.3)	0.728
3	53 (63.9)	31 (57.4)	6 (66.7)	
TNM stage, n (%)				
III B/III C	21 (25.3)	12 (22.2)	1 (11.1)	0.774
IV/Recurrent	62 (74.7)	42 (77.8)	8 (88.9)	
PD-L1 TPS, n (%)				
TPS < 1%	27 (32.5)	17 (31.5)	2 (22.2)	0.948
1% ≤ TPS ≤ 49%	23 (27.7)	19 (35.2)	3 (33.3)	
TPS ≥ 50%	24 (28.9)	14 (25.9)	3 (33.3)	
Not evaluable	9 (10.8)	4 (7.4)	1 (11.1)	
Radiotherapy, n (%)				
Yes	17 (20.5)	10 (18.5)	2 (22.2)	0.947
No	66 (79.5)	44 (81.5)	7 (77.8)	
irAEs, n (%)				
Yes	16 (19.3)	5 (9.3)	1 (11.1)	0.302
No	67 (80.7)	49 (90.7)	8 (88.9)	
PRE-ALB, n (%)				
< 3.5 g/dL	18 (21.7)	12 (22.2)	3 (33.3)	0.693
≥ 3.5 g/dL	65 (78.3)	42 (77.8)	6 (66.7)	
POST-ALB, n (%)				
< 3.5 g/dL	6 (7.2)	8 (14.8)	3 (33.3)	**0.041**
≥ 3.5 g/dL	77 (92.8)	46 (85.2)	6 (66.7)	

*Non-squamous tumor included adenocarcinoma and lymphoepithelioma-like carcinoma.

ECOG PS, Eastern Cooperative Oncology Group performance status; NOS, not otherwise specified; PD-L1, programmed cell death-protein 1; TPS, tumor proportion score. irAEs, immune-related adverse events; ALB, albumin; LIPI, lung immune prognostic index. Significant p values (<0.05) are in bold.

### 3.2 Relationship between LIPI and ORR

**Figure 1:**
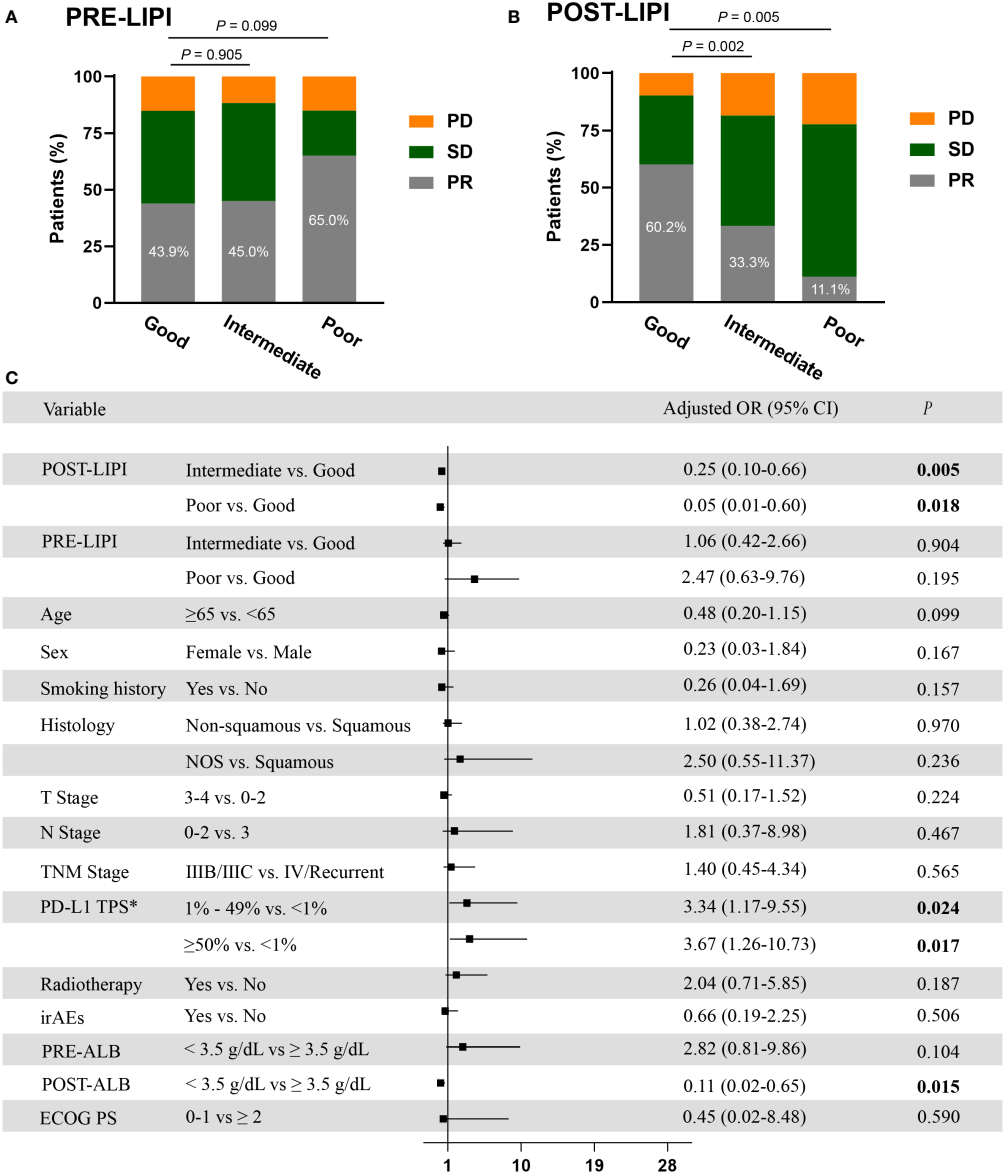
The association between LIPI and ORR. Treatment response by PRE-LIPI **(A)** and POST-LIPI **(B)** groups. Multivariate logistic regression analysis of ORR **(C)**. *Only for patients with available PD-L1 expression data. ORR, objective response rate; PD, progressive disease; SD, stable disease; PR, partial response, OR, odds ratio; LIPI, Lung Immune Prognostic Index; NOS, not otherwise specified; PD-L1, programmed cell death ligand 1; TPS, tumor proportion score; irAEs, immune-related adverse events; ALB, albumin; ECOG PS, Eastern Cooperative Oncology Group performance status.

In this study, 69 (47.3%) patients achieved objective response. ORRs were 43.9%, 45.0%, and 65.0% in good, intermediate, and poor PRE-LIPI groups, respectively. The differences between good PRE-LIPI and intermediate PRE-LIPI groups (*P* = 0.905), as well as poor PRE-LIPI group (*P* = 0.099) were not significant (**Figure 1A**). ORRs in good, intermediate, and poor POST-LIPI groups were 60.2%, 33.3%, and 11.1%, respectively. ORRs in intermediate POST-LIPI group (*P* = 0.002) and poor POST-LIPI group (*P* = 0.005) were significantly lower than those in good POST-LIPI (**Figure 1B**).

Compared with good POST-LIPI group, a significant association of intermediate POST-LIPI [odds ratio (OR), 0.33, 95% CI, 0.16-0.68; *P* = 0.002] and poor POST-LIPI (OR, 0.08, 95% CI, 0.01-0.69; *P* = 0.021) with a lower ORR was observed in univariate analysis (**Supplementary Table 2**). Additionally, in the multivariate logistic regression analysis, intermediate POST-LIPI (adjusted OR, 0.25, 95% CI, 0.10-0.66; *P* = 0.005) and poor POST-LIPI (adjusted OR, 0.05, 95% CI, 0.01-0.60; *P* = 0.018) were also significantly associated with lower ORRs. Moreover, 1% ≤ PD-L1 TPS ≤ 49% (adjusted OR, 3.34, 95% CI, 1.17-9.55; *P* = 0.024), PD-L1 ≥ 50% (adjusted OR, 3.67, 95% CI, 1.26-10.73; *P* = 0.017), and POST-ALB (adjusted OR, 0.11, 95% CI, 0.02-0.65; *P* = 0.015) were also independently associated with ORR (**Figure 1C**). No significant association was found between PRE-LIPI and ORR in univariate and multivariate logistic regression analyses (**Supplementary Table 2** and **Figure 1C**).

### 3.3 Association between LIPI and PFS

**Figure 2:**
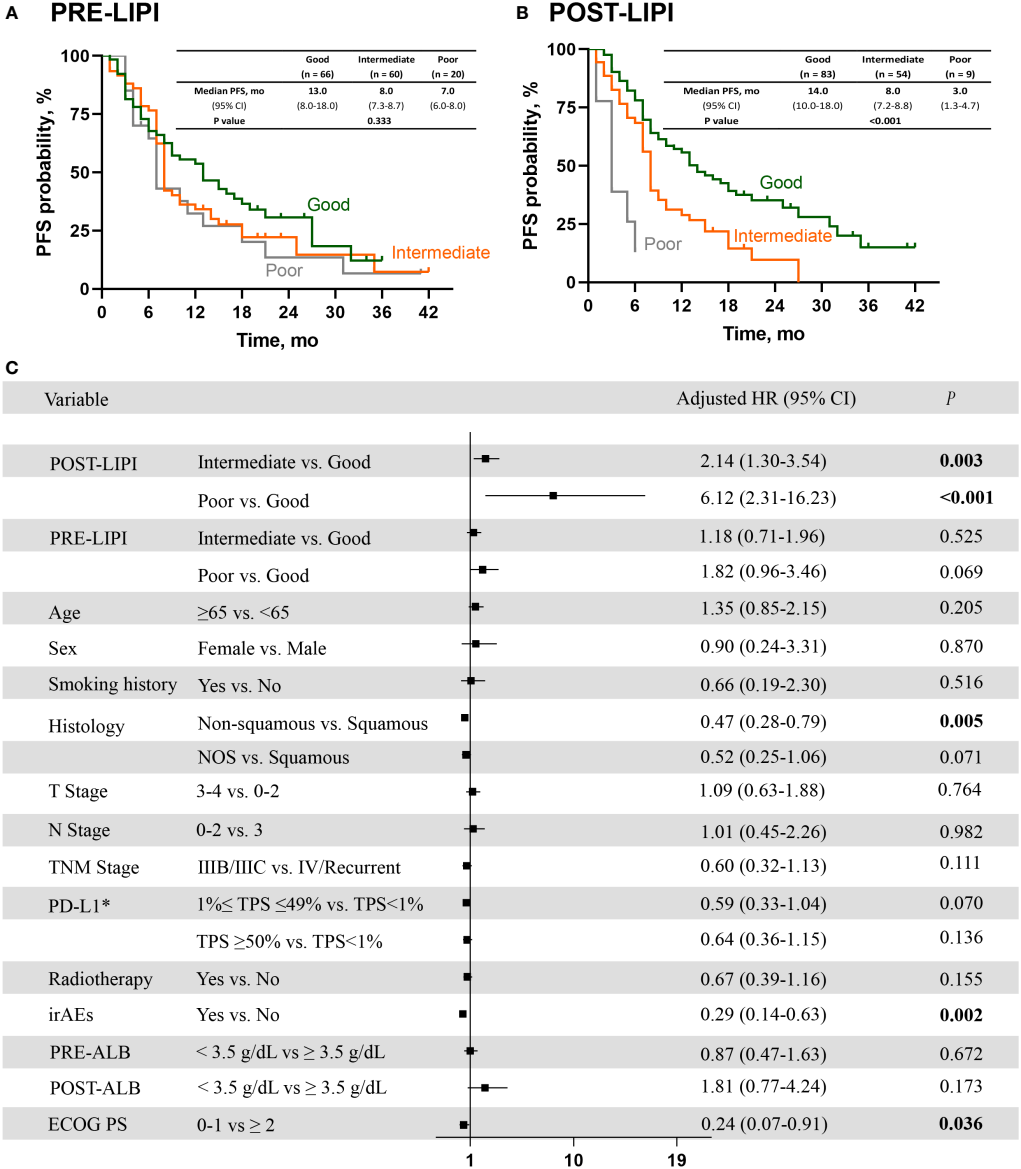
The association between LIPI and PFS. Kaplan-Meier curves for PFS according to PRE-LIPI **(A)** and POST-LIPI **(B)**. Multivariate Cox regression analysis of PFS **(C)**. *Only for patients with available PD-L1 expression data. PFS, progression-free survival; LIPI, lung immune prognostic index. HR, hazard ratio; NOS, not otherwise specified; PD-L1, programmed cell death ligand 1; TPS, tumor proportion score; irAEs, immune-related adverse events; ALB, albumin; ECOG PS, Eastern Cooperative Oncology Group performance status.

As shown in **Figure 2A**, median PFS was 13.0 months (95% CI, 8.0-18.0) versus 8.0 months (95% CI, 7.3-8.7) versus 7.0 months (95% CI, 6.0-8.0) in good PRE-LIPI group, intermediate PRE-LIPI group, and poor PRE-LIPI group, respectively (*P* = 0.333). As for the POST-LIPI status, median PFS was 14 months (95% CI, 10.0-18.0) versus 8.0 months (95% CI, 7.2-8.8) versus 3.0 months (95% CI, 1.3-4.7) in good POST-LIPI group, intermediate POST-LIPI group, and poor POST-LIPI group, respectively (*P* < 0.001) (**Figure 2B**). A significant decrease was found in PFS in intermediate POST-LIPI group (HR, 1.95; 95% CI, 1.28-2.98; *P* = 0.002) and poor POST-LIPI group (HR, 7.26; 95% CI, 3.10-17.04; *P* < 0.001) compared with that in good POST-LIPI group in the univariate analysis. No significant association between PRE-LIPI and PFS was identified (**Supplementary Table 3**).

After checking for the covariates of age, sex, smoking history, histology, T stage, N stage, TNM stage, PD-L1 expression level, radiotherapy, irAEs, ECOG PS and PRE (POST)-ALB, patients with intermediate POST-LIPI score (adjusted HR, 2.14; 95% CI, 1.30-3.54; *P* = 0.003) or poor POST-LIPI score (adjusted HR, 6.12; 95% CI, 2.31-16.23; *P* < 0.001) had a significantly shorter PFS than those with good POST-LIPI score. Moreover, non-squamous (adjusted HR, 0.47; 95% CI, 0.28-0.79; *P* = 0.005), irAEs (adjusted HR, 0.29; 95% CI, 0.14-0.63; *P* = 0.002) and ECOG PS (adjusted HR, 0.24; 95% CI, 0.07-0.91; *P* = 0.036)were also independently associated with a significantly longer PFS (**Figure 2C**).

To estimate the predictive value of PRE-LIPI and POST-LIPI, time-dependent ROC curves was drawn. The area under the curves (AUC) of POST-LIPI were numerically larger than PRE-LIPI (AUC at 1 year: 0.66>0.60, P = 0.306; AUC at 18 months: 0.67>0.57, P = 0.110; AUC at 2 years: 0.71>0.57, P = 0.167) (**Supplementary Figure 3**). The predictive power of PRE-LIPI and POST-LIPI is shown in **Supplementary Table 4**.

### 3.4 The predictive value of POST-LIPI in patients with the negative or low PD-L1 expression level

**Figure 3:**
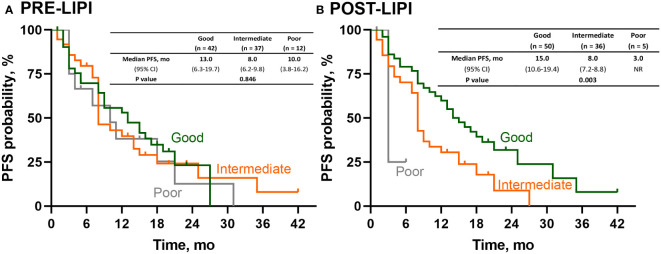
Survival analysis of PFS in patients with negative or low PD-L1 expression. Kaplan-Meier curves for PFS according to **(A)** PRE-LIPI and **(B)** POST-LIPI. PFS, progression-free survival; LIPI, lung immune prognostic index.

We further investigated the association between LIPI and PFS in patients with the negative or low PD-L1 expression level. As shown in **Figure 3A**, there was no significant association between PRE-LIPI and PFS (*P* = 0.846). Regarding POST-LIPI, patients with intermediate POST-LIPI (median PFS, 8.0 months; 95% CI, 7.2-8.8) and poor POST-LIPI (median PFS, 3.0 months; 95% CI, NR) had a significantly shorter PFS than those with good POST-LIPI (median PFS, 15.0 months; 95% CI, 10.6-19.4) (*P* = 0.003) (**Figure 3B**). According to the results of univariate analysis, compared with patients with good POST-LIPI (ORR, 58.0%), patients with intermediate POST-LIPI (ORR, 33.3%, OR, 0.36, 95% CI, 0.15-0.88; *P* = 0.026) were significantly associated with lower ORRs. However, the association between poor POST-LIPI and ORR was not found (ORR, 20.0%, OR, 0.18, 95% CI, 0.02-1.74; *P* = 0.139).

### 3.5 A predictive model combining PRE-LIPI with POST-LIPI

**Figure 4:**
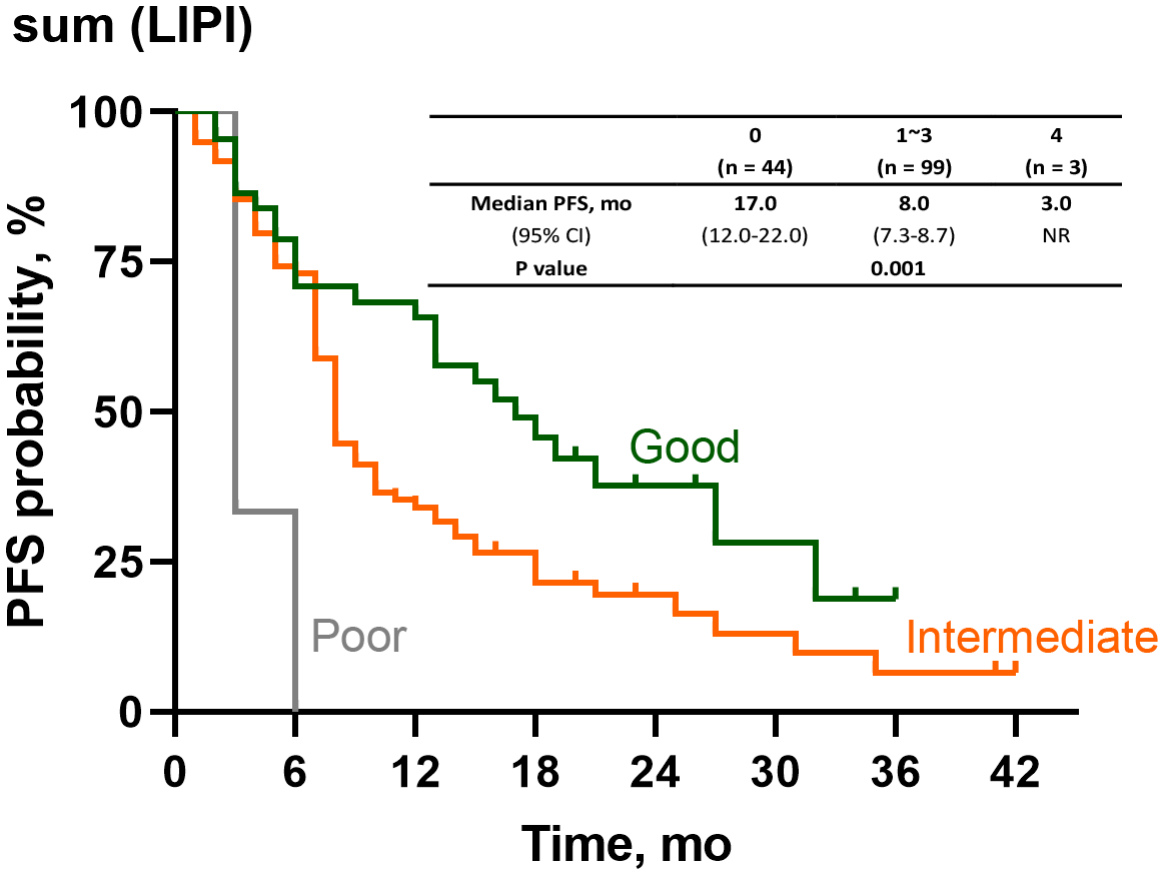
Survival analysis of PFS according to sum(LIPI). PFS, progression-free survival, LIPI, Lung Immune Prognostic Index.

After the time-dependent ROC curves had been drawn, we found that the AUC of POST-LIPI was only numerically larger than PRE-LIPI (**Supplementary Table 4**). To predict the treatment efficacy with LIPI in a better way, we analyzed the relationship between PFS and sum (LIPI) [sum(LIPI) = PRE-LIPI + POST-LIPI]. As LIPI assessed two inflammatory risk factors (dNLR > 3 and LDH level > ULN), patients who achieved 0 both at baseline (PRE-LIPI) and early in the treatment (POST-LIPI) were in [Sum(LIPI)=0] group, and other patients who achieved 2 at both the two time points were in [Sum(LIPI)=4] group, while the remaining patients were in [Sum(LIPI)=1~3] group. Compared with sum (LIPI)=0 group (median PFS, 17.0 months), those patients with sum (LIPI)=1~3 (median PFS, 8.0 months, HR, 1.73;95%CI, 1.09-2.74;P=0.019) or with sum (LIPI)=4 (median PFS, 3.0 months, HR, 7.29;95%CI, 2.12-25.10;P=0.002) had a significantly shorter PFS (**Figure 4**).

In the analyses of time-dependent ROC curve, the AUC of sum (LIPI) were significantly larger than PRE(LIPI) (AUC at 1 year: 0.69>0.60, P = 0.003; AUC at 18 months: 0.66>0.57; P < 0.001; AUC at 2 years: 0.68>0.57; P = 0.003) (**Supplementary Figure 4**). The predictive value of sum(LIPI) is shown in **Supplementary Table 4**.

### 3.6 LIPI early dynamic predicts ORR and PFS

In order to analyze the predictive value of LIPI dynamic, we classified good PRE-LIPI group into superior PRE-LIPI group and intermediate PRE-LIPI group as well as poor PRE-LIPI group into inferior PRE-LIPI group. The designated of LIPI dynamic was shown in **Supplementary Figure 5**. Patients with higher POST-LIPI than PRE-LIPI were in LIPI negative change group. Those with lower POST-LIPI than PRE-LIPI were in LIPI positive change group. And the patients have POST-LIPI equal to PRE-LIPI were in LIPI no change group. We have done logistic regression analysis and Cox regression analysis In superior PRE-LIPI group, compare with no change group, those with negative change LIPI had significant lower ORR (OR, 0.17, 95% CI, 0.05-0.58; P = 0.005) and shorter PFS (HR, 2.31; 95% CI, 1.23-4.36; P = 0.010). In inferior PRE-LIPI, compare with positive change group, those with no change LIPI had significant lower ORR (OR, 0.32, 95% CI, 0.13-0.80; P = 0.015) and shorter PFS (HR, 1.98; 95% CI, 1.15-3.40; P = 0.013).

## 4 Discussion

In the present study, we demonstrated that a higher POST-LIPI score was associated with a lower ORR and a shorter PFS in NSCLC patients receiving first-line PD-1 inhibitor combined with chemotherapy. In patients with the negative or low PD-L1 expression level, a higher POST-LIPI score was associated with worse efficacy of the combination therapy. In addition, a higher sum (LIPI) was correlated with a shorter PFS. The above-mentioned results may indicate that LIPI can be used for continuous assessment of the treatment efficacy during chemoimmunotherapy.

Our results suggested that for NSCLC patients receiving first-line PD-1 inhibitor in combination with chemotherapy, a higher POST-LIPI score was associated with worse treatment efficacy. LIPI is an index calculated based on peripheral blood count and LDH level, which are commonly measured in the majority of hospitals. Therefore, LIPI is an easily accessible biomarker. Compared with PD-L1, the detection of LIPI is noninvasive, thus, the application of LIPI in clinical practice can reduce patient suffering and increase patient compliance. A non-linear relationship has been reported between bTMB and prognosis in patients receiving immunotherapy (18), therefore, there are still difficulties in determining cutoff value of bTMB, whereas LIPI is clinically actionable. In addition, the results of the present study suggested that PRE-LIPI is not predictive of the chemoimmunotherapy efficacy. The possible reason might be that patients in the same PRE-LIPI subgroup have different POST-LIPI scores. Our results showed that in each PRE-LIPI subgroup, a higher POST-LIPI score was significantly correlated with a shorter PFS (**Supplementary Figure 2**). This finding suggested that after two cycles of treatment, patients’ systemic inflammatory status might change, and LIPI in the early-stage of treatment is more reflective of patients’ inflammatory status receiving the combination therapy than LIPI at baseline. In addition, the results of this study showed that sum (LIPI) was correlated with patients’ PFS. The AUC of sum (LIPI) was significantly larger than PRE-LIPI. Therefore, continuous assessment of LIPI in the early-stage of treatment might be effective and necessary since it might help clinicians to predict efficacy and adjust treatments in time.

Our results suggested that in patients with the negative or low PD-L1 expression level, higher POST-LIPI scores were associated with worse treatment efficacy. For patients with the PD-L1 expression level < 50% and without driver mutations, chemoimmunotherapy is one of the most common first-line therapies. However, previous clinical trials have shown that its benefit is not remarkable in those with the negative or low PD-L1 expression level compared with those with the high PD-L1 expression level (PD-L1 expression level ≥ 50%) (6, 19). This suggested that there is a need to identify patients who can benefit from the combination treatment in the population with the negative or low PD-L1 expression level. In the present study, POST-LIPI score was correlated with the therapeutic efficacy in patients with the negative or low PD-L1 expression level. Moreover, POST-LIPI score was not associated with PD-L1 expression level, suggesting that the relationship between POST-LIPI score and the treatment efficacy was not mediated by PD-L1 expression level. Therefore, POST-LIPI score might also play a role in predicting the therapeutic efficacy in patients with the negative or low PD-L1 expression level.

The present study had some limitations. First, this was a retrospective study. Thus, data related to the LIPI score after the first administration were unavailable. Second, due to the long median survival of patients receiving chemoimmunotherapy (27, 28), patients’ overall survival was not assessed in this study. Third, the study population was relatively small. Hence, a larger prospective study including a control group is warranted to validate our results in the future.

In conclusion, it was found that a higher POST-LIPI score was correlated with a lower ORR and a shorter PFS in NSCLC patients treated with first-line PD-1 inhibitor plus chemotherapy. Furthermore, POST-LIPI score was correlated with the efficacy of the combination therapy in patients with the negative or low PD-L1 expression level. Moreover, sum (LIPI) might be a potential predictive marker for PFS. Therefore, continues assessment of LIPI might be an effective approach to predict therapeutic efficacy in NSCLC patients receiving chemoimmunotherapy.

## Data availability statement

The datasets presented in this article are not readily available because our data came from unfinished projects. Requests to access the datasets should be directed to zxychest0109@163.com.

## Ethics statement

This study was approved by the ethics committee and institutional review board of Shanghai Chest Hospital (Shanghai, China).

## Author contributions

Conceptualization, AX, and JX. Literature Search, SW and BH. Figures, RZ, JL and TC. Data Collection and Analysis, WZ, YL, and XiZ. Writing-Original Draft, AX. Writing—review and editing, WN, HZ and XuZ. All authors contributed to the article and approved the submitted version.
