# Supplementary material for: On-treatment lung immune prognostic index is predictive for first-line PD-1 inhibitor combined with chemotherapy in patients with non-small cell lung cancer
Source: Front Immunol. 2023 May 25;14:1173025. doi: 10.3389/fimmu.2023.1173025 (PMC10247997; doi:10.3389/fimmu.2023.1173025)

Supplementary Table 1. The correlation of PRE-LIPI with other patients’ characteristics.

| Characteristics | Good  PRE-LIPI  (n = 66) | Intermediate  PRE-LIPI  (n = 60) | Poor  PRE-LIPI  (n = 20) | *P* |
| --- | --- | --- | --- | --- |
| Age, n (%) |  |  |  |  |
| <65 | 30 (45.5) | 32 (53.3) | 10 (50.0) | 0.724 |
| ≥65 | 36 (54.5) | 28 (46.7) | 10 (50.0) |  |
| Sex, n (%) |  |  |  |  |
| Male | 51 (77.3) | 49 (81.7) | 18 (90.0) | 0.481 |
| Female | 15 (22.7) | 11 (18.3) | 2 (10.0) |  |
| Smoking history, n (%) |  |  |  |  |
| Never | 17 (25.8) | 16 (26.7) | 3 (15.0) | 0.604 |
| Former/Current | 49 (74.2) | 44 (73.3) | 17 (85.0) |  |
| ECOG PS |  |  |  |  |
| 0-1 | 66 (100) | 56 (93.3) | 20 (100) | 0.076 |
| ≥ 2 | 0 (0) | 4 (6.7) | 0 (0) |  |
| Histology, n (%) |  |  |  |  |
| Squamous | 22 (33.3) | 22 (36.7) | 5 (25.0) | 0.231 |
| Non-squamous* | 38 (57.6) | 31 (51.7) | 9 (45.0) |  |
| NOS | 6 (9.1) | 7 (11.7) | 6 (30.0) |  |
| T stage, n (%) |  |  |  |  |
| 0-2 | 46 (69.7) | 36 (60.0) | 13 (65.0) | 0.491 |
| 3-4 | 20 (30.3) | 24 (40.0) | 7 (35.0) |  |
| N stage, n (%) |  |  |  |  |
| 0-2 | 31 (47.0) | 18 (30.0) | 7 (35.0) | 0.141 |
| 3 | 35 (53.0) | 42 (70.0) | 13 (65.0) |  |
| TNM stage, n (%) |  |  |  |  |
| Ⅲ B/Ⅲ C | 16 (24.2) | 17 (28.3) | 1 (5.0) | 0.083 |
| Ⅳ/Recurrent | 50 (75.8) | 43 (71.7) | 19 (95.0) |  |
| PD-L1 TPS, n (%) |  |  |  |  |
| TPS < 1% | 22 (33.3) | 18 (30.0) | 6 (30.0) | 0.950 |
| 1% ≤ TPS ≤ 49% | 20 (30.3) | 19 (31.7) | 6 (30.0) |  |
| TPS ≥ 50% | 16 (24.2) | 18 (30.0) | 7 (35.0) |  |
| Not evaluable | 8 (12.1) | 5 (8.3) | 1 (5.0) |  |
| Radiotherapy, n (%) |  |  |  |  |
| Yes | 14 (21.2) | 10 (16.7) | 5 (25.0) | 0.656 |
| No | 52 (78.8) | 50 (83.3) | 15 (75.0) |  |
| irAEs, n (%) |  |  |  |  |
| Yes | 8 (12.1) | 11 (18.3) | 3 (15.0) | 0.626 |
| No | 58 (87.9) | 49 (81.7) | 17 (85.0) |  |
| PRE-ALB, n (%) |  |  |  |  |
| < 3.5 g/dL | 9 (13.6) | 17 (28.3) | 7 (35.0) | **0.042** |
| ≥ 3.5 g/dL | 57 (86.4) | 43 (71.7) | 13 (65.0) |  |
| POST-ALB, n (%) |  |  |  |  |
| < 3.5 g/dL | 4 (6.2) | 9 (15.0) | 4 (20.0) | 0.111 |
| ≥ 3.5 g/dL | 61 (93.8) | 51 (85.0) | 16 (80.0) |  |

*Non-squamous tumor included adenocarcinoma and lymphoepithelioma-like carcinoma.

Abbreviations: ECOG PS, Eastern Cooperative Oncology Group performance status; NOS, not otherwise specified; PD-L1, programmed cell death-protein 1; TPS, tumor proportion score. irAEs, Immune-related Adverse Events; ALB, Albumin; LIPI, lung immune prognostic index.

Supplementary Table 2. Univariate analysis for ORR.

| Variable | OR (95% CI) | *P* |
| --- | --- | --- |
| Age  (≥65 vs. <65) | 0.58 (0.30-1.11) | 0.100 |
| Sex  (Female vs. Male) | 0.96 (0.42-2.19) | 0.922 |
| Smoking history  (Yes vs. No) | 0.86 (0.41-1.84) | 0.705 |
| ECOG PS  (0-1 vs ≥2) | 0.89 (0.12-6.52) | 0.911 |
| Histology |  |  |
| (Non-squamous* vs. Squamous) | 1.33 (0.65-2.74) | 0.433 |
| (NOS vs Squamous) | 1.20 (0.41-3.48) | 0.737 |
| T stage  (3-4 vs 0-2) | 0.99 (0.50-1.95) | 0.972 |
| N stage  (0-2 vs 3) | 2.16 (0.69-6.79) | 0.188 |
| TNM stage  (ⅢB/ⅢC vs. Ⅳ/Recurrent) | 1.38 (0.63-3.00) | 0.418 |
| PD-L1 TPS** |  |  |
| (1% ≤ TPS ≤ 49% vs. TPS < 1%) | 2.13 (0.92-4.94) | 0.077 |
| (TPS ≥ 50% vs. TPS < 1%) | 2.18 (0.92-5.15) | 0.076 |
| Radiotherapy  (Yes vs. No) | 1.49 (0.66-3.37) | 0.342 |
| irAEs  (Yes vs. No) | 0.74 (0.29-1.85) | 0.518 |
| PRE-ALB  (< 3.5 g/dL vs ≥ 3.5 g/dL) | 1.25 (0.57-2.71) | 0.578 |
| POST-ALB  (< 3.5 g/dL vs ≥ 3.5 g/dL) | 0.31 (0.10-0.99) | **0.049** |
| PRE-LIPI |  |  |
| (Intermediate vs. Good) | 1.04 (0.52-2.11) | 0.905 |
| (Poor vs Good) | 2.37 (0.84-6.70) | 0.104 |
| POST-LIPI |  |  |
| (Intermediate vs. Good) | 0.33 (0.16-0.68) | **0.002** |
| (Poor vs Good) | 0.08 (0.01-0.69) | **0.021** |

*Non-squamous tumor included adenocarcinoma and lymphoepithelioma-like carcinoma.

**Only for patients with available PD-L1 expression data.

Abbreviations: ORR, objective response rate; OR, odds ratio; CI, confidence interval; ECOG PS, Eastern Cooperative Oncology Group performance status; NOS, not otherwise specified; PD-L1, programmed cell death-protein 1; TPS, tumor proportion score; irAEs; immune-related Adverse Events; ALB, albumin; LIPI, lung immune prognostic index.

Supplementary Table 3. Univariate analysis for PFS.

| Variable | HR (95% CI) | *P* |
| --- | --- | --- |
| Age  (≥65 vs. <65) | 1.12 (0.75-1.66) | 0.578 |
| Sex  (Female vs. Male) | 0.93 (0.55-1.55) | 0.766 |
| Smoking history  (Yes vs. No) | 1.07 (0.66-1.74) | 0.794 |
| ECOG PS  (0-1 vs ≥2) | 0.33 (0.12-0.89) | **0.029** |
| Histology |  |  |
| (Non-squamous* vs. Squamous) | 0.56 (0.36-0.86) | **0.009** |
| (NOS vs Squamous) | 0.90 (0.50-1.63) | 0.730 |
| T stage  (3-4 vs 0-2) | 1.38 (0.92-2.09) | 0.123 |
| N stage  (0-2 vs 3) | 0.98 (0.51-1.90) | 0.953 |
| TNM stage  (ⅢB/ⅢC vs. Ⅳ/Recurrent) | 0.86 (0.53-1.39) | 0.531 |
| PD-L1 TPS** |  |  |
| (1% ≤ TPS ≤ 49% vs. TPS < 1%) | 0.70 (0.42-1.15) | 0.159 |
| (TPS ≥ 50% vs. TPS < 1%) | 0.75 (0.45-1.27) | 0.282 |
| Radiotherapy  (Yes vs. No) | 0.70 (0.43-1.13) | 0.144 |
| irAEs  (Yes vs. No) | 0.41 (0.21-0.78) | **0.007** |
| PRE-ALB  (< 3.5 g/dL vs ≥ 3.5 g/dL) | 1.13 (0.72-1.80) | 0.593 |
| POST-ALB  (< 3.5 g/dL vs ≥ 3.5 g/dL) | 1.56 (0.85-2.87) | 0.150 |
| PRE-LIPI |  |  |
| (Intermediate vs. Good) | 1.26 (0.82-1.94) | 0.296 |
| (Poor vs Good) | 1.45 (0.82-2.56) | 0.197 |
| POST-LIPI |  |  |
| (Intermediate vs. Good) | 1.95 (1.28-2.98) | **0.002** |
| (Poor vs Good) | 7.26 (3.10-17.04) | **<0.001** |

*Non-squamous tumor included adenocarcinoma and lymphoepithelioma-like carcinoma.

**Only for patients with available PD-L1 expression data.

Abbreviations: ORR, objective response rate; OR, odds ratio; CI, confidence interval; ECOG PS, Eastern Cooperative Oncology Group performance status; NOS, not otherwise specified; PD-L1, programmed cell death-protein 1; TPS, tumor proportion score; irAEs; immune-related Adverse Events; ALB, albumin; LIPI, lung immune prognostic index.

Supplementary Table 4. The predictive power of PRE-LIPI, POST-LIPI and sum(LIPI).

|  | time | AUC  (95% CI) | sensitivity  (95% CI) | specificity  (95% CI) | PPV  (95% CI) | NPV  (95% CI) |
| --- | --- | --- | --- | --- | --- | --- |
| PRE-LIPI | at 1 year | 0.60  (0.51-0.69) | 0.64  (0.52-0.75) | 0.56  (0.43-0.70) | 0.65  (0.53-0.76) | 0.55  (0.42-0.68) |
|  | at 18 months | 0.57  (0.46-0.67) | 0.59  (0.48-0.69) | 0.55  (0.37-0.73) | 0.73  (0.61-0.84) | 0.39  (0.25-0.53) |
|  | at 2 years | 0.57  (0.41-0.74) | 0.58  (0.48-0.69) | 0.58  (0.30-0.87) | 0.81  (0.68-0.94) | 0.31  (0.16-0.46) |
| POST-LIPI | at 1 year | 0.66  (0.58-0.74) | 0.56  (0.44-0.68) | 0.75  (0.63-0.86) | 0.73  (0.61-0.85) | 0.58  (0.46-0.69) |
|  | at 18 months | 0.67  (0.58-0.75) | 0.51  (0.40-0.62) | 0.81  (0.66-0.95) | 0.84  (0.73-0.96) | 0.44  (0.32-0.56) |
|  | at 2 years | 0.71  (0.62-0.80) | 0.50  (0.40-0.61) | 0.92  (0.76-100) | 0.95  (0.85-100) | 0.37  (0.25-0.50) |
| sum  (LIPI) | at 1 year | 0.69  (0.60-0.77) | 0.83  (0.74-0.91) | 0.47  (0.34-0.61) | 0.66  (0.56-0.76) | 0.68  (0.54-0.83) |
|  | at 18 months | 0.66  (0.55-0.77) | 0.77  (0.67-0.86) | 0.48  (0.30-0.66) | 0.75  (0.66-0.85) | 0.50  (0.33-0.67) |
|  | at 2 years | 0.68  (0.54-0.82) | 0.74  (0.65-0.84) | 0.50  (0.21-0.79) | 0.82  (0.71-0.93) | 0.39  (0.19-0.58) |

Supplementary Figure 1

Patients with lung cancer (4178)

pathologically diagnosed as SCLC(626)

with stage Ⅰ-ⅢA NSCLC(1563)

with sensitizing EGFR/ALK/ROS1 mutation (616)

Stage ⅢB-Ⅳ or recurrent NSCLC patients without sensitizing EGFR/ALK/ROS1 mutation (1373)

Not received first-line PD-1 inhibitor combined with chemotherapy (1185)

Stage ⅢB-Ⅳ NSCLC or recurrent patients treated with first-line PD-1 inhibitor plus chemotherapy with available data (146)

Data not available (22)

Exposed to infection or antibiotics within 7 days before blood draw (3)

Received immune checkpoint inhibitor-based neoadjuvant therapy (17)

Supplementary Figure 2


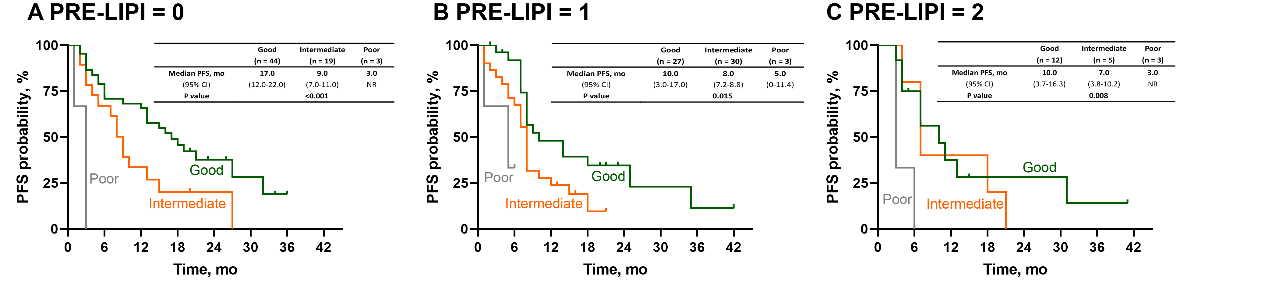


Supplementary Figure 3


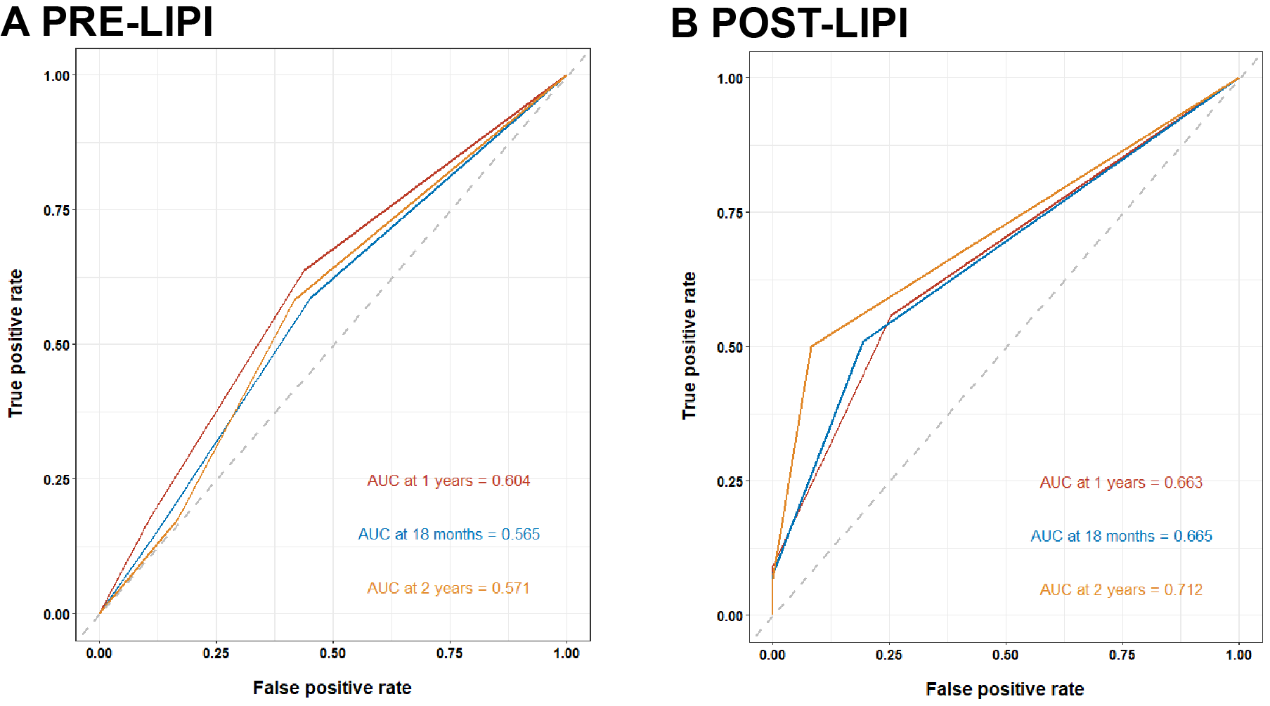


Supplementary Figure 4


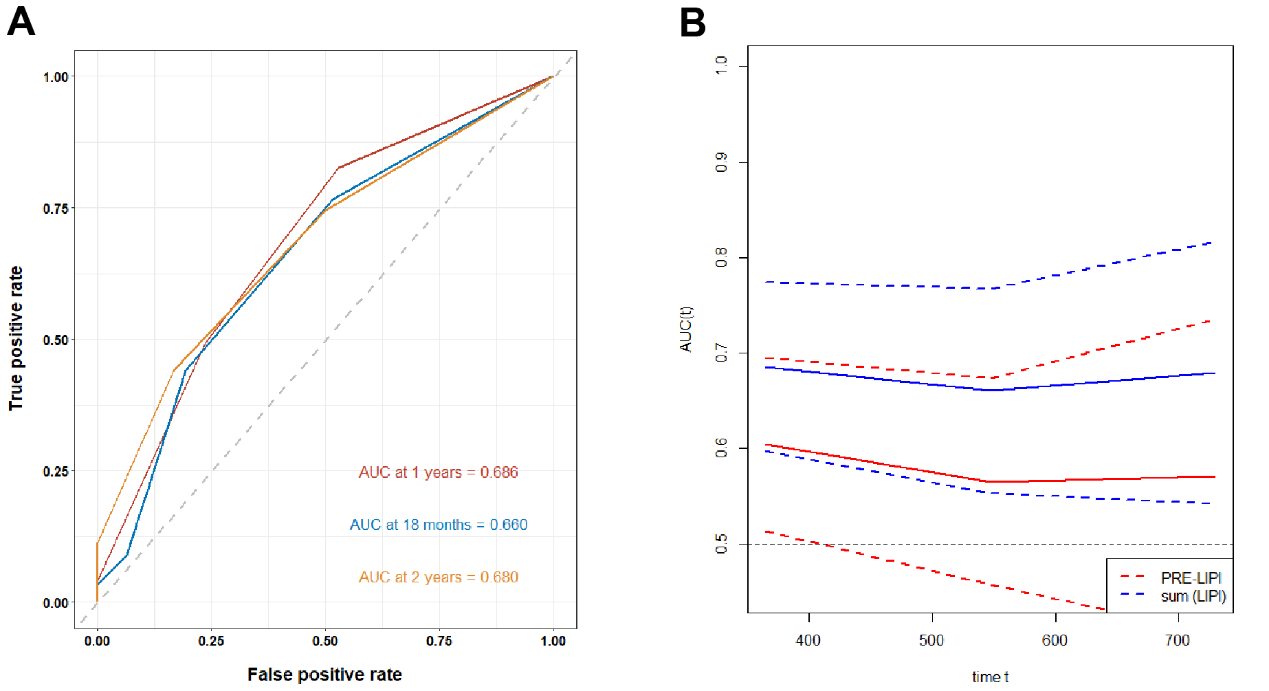


Supplementary Figure 5


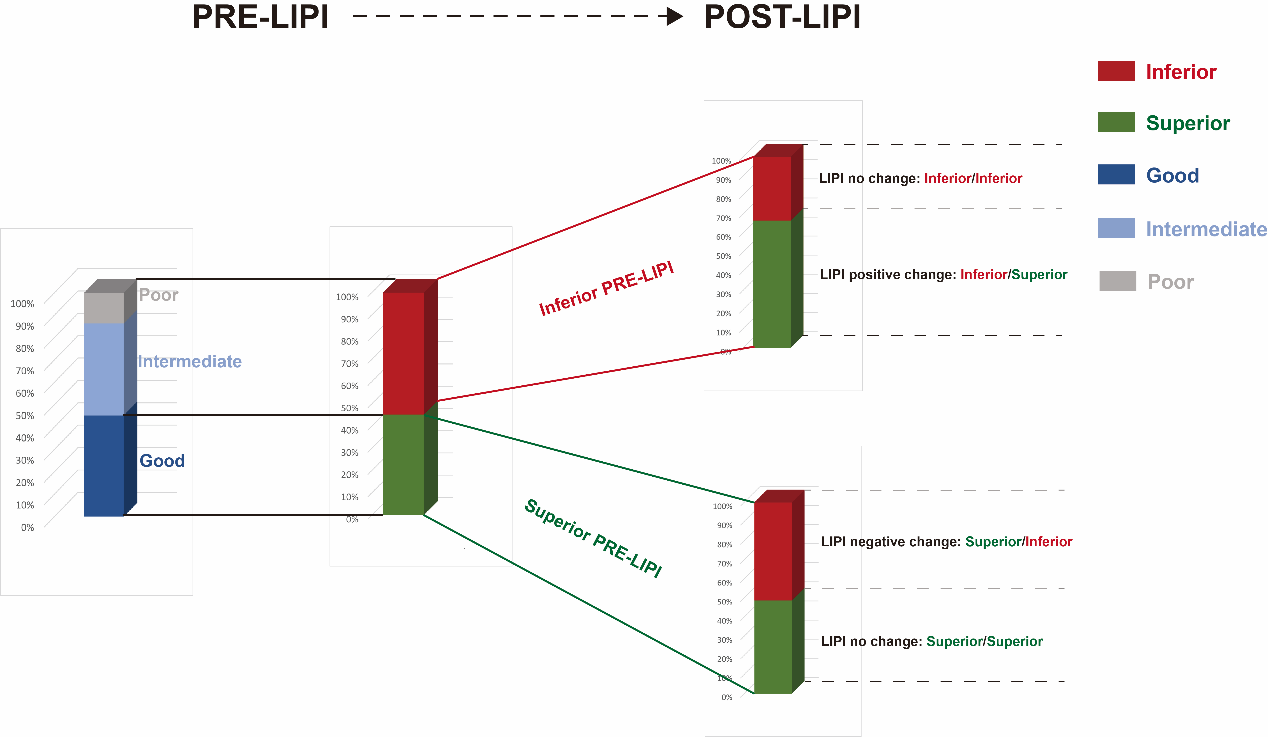

Supplement: Supplementary file 1 [file DataSheet_1.docx]
